# Evaluation of Different Commercial Sensors for the Development of Their Automatic Irrigation System

**DOI:** 10.3390/s24237468

**Published:** 2024-11-22

**Authors:** Sandra Millán, Cristina Montesinos, Carlos Campillo

**Affiliations:** Centro de Investigaciones Científicas y Tecnológicas de Extremadura (CICYTEX), Finca La Orden, Regional Government of Extremadura, Highway A-V, Km 372, 06187 Guadajira, Badajoz, Spain; cristina.montesinos@juntaex.es (C.M.); carlos.campillo@juntaex.es (C.C.)

**Keywords:** capacitance, frequency domain reflectometry, impedance, time domain reflectometry, tensiometers, irrigation, calibration

## Abstract

Reliable soil moisture information is essential for accurate irrigation scheduling. A wide range of soil moisture sensors are currently available on the market, but their performance needs to be evaluated as most sensors are calibrated under limited laboratory conditions. The aim of this study was to evaluate the performance of six commercially available moisture sensors (HydraProbe, Teros 10, Teros 11, EnviroPro, CS616 and Drill & Drop) and three tensiometers (Irrometer RSU-C-34, Teros 32 and Teros 21) and to establish calibration equations for a typical sandy soil of the Doñana National Park (Huelva, Spain). The calibration process for soil moisture sensors indicated differences between factory and corrected equations. All tested sensors improved with adjustments made to the factory calibration, except for the HydraProbe sensor which had a more accurate factory equation for a sandy soil. Among the various sensors tested, the Teros 10, Teros 11, and HydraProbe were found to be the easiest to install, typically positioned with an auger to prevent preferential pathways and ensure adequate sensor-soil contact. For tensiometers, the Teros 32 sensor requires specialized labor for its correct installation, as it must be positioned at a specific angle and maintained with distilled water. All tensiometers need a stabilization period after installation.

## 1. Introduction

Agricultural intensification, population growth and climate change have put pressure on available water resources [1]. The agricultural sector is the largest global consumer of freshwater in the world. According to the United Nations World Water Development Report 2020 [2], croplands are responsible for 69% of water withdrawals. Therefore, there is an urgent need to adopt efficient irrigation systems to reduce water consumption in agriculture, mitigate climate change and improve farm profitability.

One way to solve this problem is to reduce inefficiencies in irrigation management. Automated drip irrigation systems are an innovative irrigation management tool that allows the continuous monitoring of soil moisture in real time, maintaining specified soil water levels so that the plant does not suffer stress and enabling adjusted harvests in terms of quality and quantity. Soil moisture is a key factor in the soil-plant-atmosphere water cycle and is a critical parameter for understanding soil water movement [3]. Soil moisture sensors (SMSs) have long been used in agriculture for accurate irrigation scheduling [4], as they measure soil moisture continuously, nondestructively and at different soil depths.

Soil moisture (SM) is the amount of water present in the soil pores and can be measured using direct methods (soil sampling) or indirect methods (soil moisture sensing). Direct methods such as gravimetric or volumetric techniques directly measure the moisture content present in the soil [5]. The gravimetric method involves calculation of soil moisture according to the difference in weight between the soil in natural conditions and after being dried in an oven at 105 °C to constant weight. The volumetric water content can be calculated by multiplying the gravimetric water content by the bulk soil density [6]. While these methods are highly accurate and used to calibrate soil moisture sensors [7,8,9,10], they are destructive techniques which require a lot of labor to collect the samples and do not provide real-time monitoring of soil moisture.

Indirect methods of soil moisture measurement are based on measuring some soil characteristic in response to soil moisture changes. Such methods include the deployment of a variety of sensors that can be classified by the technology they use: neutron probes [11], tensiometers [12], gypsum blocks [13], granular matrix sensors [14], resistive probes [15], time domain reflectometry (TDR) sensors [16], frequency domain reflectometry (FDR) sensors [3], time domain transmission (TDT) sensors and coaxial impedance dielectric reflectometry sensors [17]. Of the methods mentioned above, FDR and TDR sensors are the most widely used at present to estimate the SM.

Conversion or calibration equations provided by manufacturers transform raw sensor measurements into SM [18]. Typically, soil moisture sensors are calibrated under limited laboratory conditions, which are not always appropriate for field conditions. The values obtained by soil moisture sensors are affected by site characteristics (soil type and soil moisture), soil homogeneity, and the presence of stones and roots [19,20]. Sensor manufacturers provide generic equations for mineral and organic soils but acknowledge that SMSs are more accurate when soil-specific calibration equations are used [21]. The performance of a given SMS can be increased by 2–3% if soil-specific calibration is performed [22]. Therefore, to improve the accuracy and working range of a soil moisture sensor, it is essential to perform soil-specific calibration to match the particular plot conditions.

Currently, there is a wide variety of SMSs on the market, which means that farmers must have technical knowledge when choosing a suitable sensor for each soil type, land use and irrigation system used. This, coupled with the location, installation and adjustment of the sensor to a specific area, ensures the success of proper sensor-based decision making.

One of the objectives of the Life4Doñana project was to demonstrate and promote the adoption of an automatic on-demand irrigation system in strawberry cultivation in an intensive system under plastic. This automatic irrigation system was to increase irrigation efficiency, reduce water withdrawals and nutrient pollution in the area of influence of the Doñana National Park (Almonte, Spain). For this reason, it is necessary to identify the sensor that is best suited to the particular conditions of the study area.

The objective of this work is to evaluate the performance of six moisture sensors (HydraProbe, Teros 10, Teros 11, EnviroPro, CS616 and Drill & Drop) and three tensiometers (Irrometer RSU-C-34, Teros 32 and Teros 21) available on the market and to establish calibration equations for a typical sandy soil used for strawberry production in the Doñana National Park (Huelva, Spain). This objective was established as part of the LIFE4DOÑANA project to identify the sensors best suited to an on-demand automated irrigation system for the soil conditions and crop needs of the Doñana environment in order to obtain greater economic, productive and environmental benefits.

## 2. Materials and Methods

### 2.1. Moisture Sensor Description

#### 2.1.1. Impedance

The Stevens HydraProbe II sensor is based on a principle called coaxial dielectric impedance reflectometry. The HydraProbe operates at 50 MHz and measures real (Ꜫ) and imaginary (Ꜫ’) dielectric permittivities, helping to reduce measurement errors in the measurement caused by the effects of temperature, salinity and soil type. The raw signal outputs are five analog dc voltages which are used to calculate Ꜫ, Ꜫ’, bulk electrical conductivity, and temperature [23]. The general calibration equation provided by the manufacturer is as follows:

(1) $$\mathrm{SM}=0.109\sqrt{\varepsilon }-0.179$$

#### 2.1.2. Frequency Domain Reflectometry (FDR)

FDR sensors are based on the resonance characteristic of RLC (resistor, inductor and capacitor) circuits in which a capacitor is formed by two electrodes and surrounding soil [24]. These sensors measure the volumetric content indirectly through the apparent dielectric constant of the soil, but by measuring the time it takes to charge a capacitor that uses the soil as a dielectric. They are therefore also called capacitive sensors. They operate at frequencies between 10 and 100 MHz. The equations provided by the manufacturers for mineral soils of the different FDR sensors are as follows:

Teros 10:

SM = 1.895 × 10^−^¹⁰ × RAW^3^ − 1.222 × 10^−^⁶ × RAW² + 2.855 × 10^−3^ × RAW − 2.154 (2)

Teros 11:

SM = 3.879 × 10^−^⁴ × RAW − 0.6956 (3)

Drill & Drop:

SM = (A × RAWᴮ) + C; A = 0.012000; B = 1; C = 0.416000 (for sandy soils; ρ = 1.5 g/cm^3^) (4)

EnviroPro:

(5) SM=8×ln(x)−7(for sandy soils)

where RAW is the raw sensor output; A, B and C are coefficients for a range of sand soil and x is the dielectric constant

#### 2.1.3. Time Domain Reflectometry (TDR)

The principle behind the TDR method is the determination of the velocity of propagation and return (or reflection) of an electromagnetic wave in a medium [25]. The presence of water in the soil decreases the speed at which the electromagnetic wave travels, which allows the measurement of soil water content. The general calibration equation provided by the manufacturer for mineral soils is as follows:

CS616:

SM = −0.0663 − 0.0063 × period + 0.0007 × period²(6)

The characteristics of the study sensors are shown in Table 1.

### 2.2. Tensiometers

The main characteristics of the tensiometers are detailed in Table 2.

### 2.3. Determination of Field Capacity, Permanent Wilting Point and Water Available to the Plant

To determine the field capacity (CC) of the soil, the permanent wilting point (PWP) in the soil and the water available to the plant (WAP), the HYPROP (UMS, München, Germany) method as described by Schindler et al. [26] was used in combination with the WP4C dewpoint potentiometer (Decagon Devices, Pullman, WA, USA).

An undisturbed soil sample was collected from one of the six pilot farms that form part of the LIFE4DOÑANA project in the area surrounding the Doñana Park, Spain. The selected farm on which the samples were taken was that with a soil texture closest to the mean and median of all the farms (latitude 37°15′68.98″ N, longitude 6°48′19.63″ W datum WGS8). Soil texture is sandy, with an average 84% sand content, 8% silt content and 8% clay content. The sample was taken with a 250 cm^3^ stainless steel ring on 22 January 2021 at a depth of 0.2 m in the ridge under the plant. In the laboratory, the sample was taken to capillary saturation. The sample was then analyzed with HYPROP according to the manufacturer’s specifications [27]. The HYPROP is a device that uses the simplified evaporation method [28]. The procedure used by HYPROP is based on the direct measurement of the water potential in the soil by inserting small tensiometers into the sample while the moisture content of the sample is progressively evaporated. The HYPROP device works at suctions of between 0 and −85 kPa.

A sample of the disturbed soil was also taken from the same farm at 0.2 m for analysis with a WP4C dew point hygrometer (Table 3) that measures water potential pointwise over a range from −0.1 MPa to −300 MPa. For the WP4C system, samples were sieved and wetted to varying degrees [29]. Combining the information obtained from Hyprop and WP4, values for CC, PWP and WAP were obtained using the HYPROP-FIT software (version 4.2.2, METER Group) adjusted to the Fredlung-Xing PDI model (Table 3).

### 2.4. Laboratory Experiment

**Figure 1:**
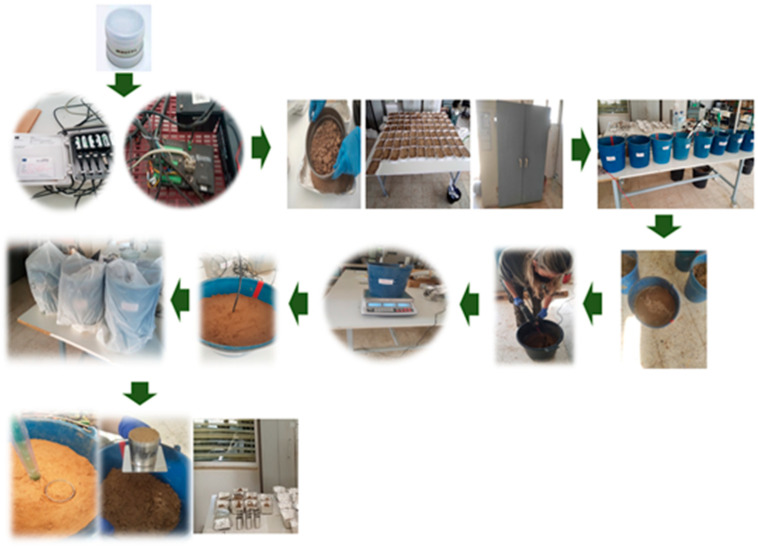
Schematic diagram of the process to carry out sensor calibration.

To determine the calibration curve for each of the sensors, 172 kg of soil were collected from the study plot. This soil was then taken to a laboratory in which the ambient temperature was at 20 °C. The process carried out was as follows (Figure 1):

1. Another soil sample was collected on 22 January, 2021 to determine the bulk density (ρa) of the study area. A cylinder of known volume (250 cm^3^) was used and dried in an oven at 105 °C to constant weight (ρa = 1.56 g/cm^3^) [30].
2. The Irrometer, HydraProbe, EnviroPro, CS616 and Drill & Drop sensors were connected to a CR1000 datalogger (Campbell Scientific Inc., Logan, UT, USA). The Teros 10, Teros 11, Teros 21 and Teros 32 sensors were connected to a ZL6 datalogger (METER Group, Inc., Pullman, USA). The information stored in both dataloggers was downloaded daily.
3. The soil was then prepared to start the test. The soil was sieved (2–5 mm) and then put on aluminum trays and placed in an oven at 105 °C until a constant weight was reached to ensure a moisture content which we call zero.
4. Preparation of the containers in which the calibration process will be carried out. For all the sensors, a weight of 12 kg of processed soil was used, except for the CS616 sensor, which used a 9 kg container as the format of this sensor requires a different container.
5. A volume of water was added daily to the total soil (0%, 2%, 4%, 6%, 7%, 8%, 9%, 10%, 11%, 12% and 13%) until field capacity was reached.
6. The soil was then thoroughly mixed and left to stand for 24 h so that the water was distributed by capillarity throughout the volume of soil.
7. The soil was then placed in the containers. Filling was carried out in such a way that a certain amount (weight) of soil occupied a certain volume of the container. For this purpose, the container was compacted as it was filled. In short, the aim was to guarantee a constant bulk density value close to that of the soil under natural conditions.
8. Subsequently, each of the containers was weighed.
9. The sensors were then inserted vertically into the soil and the readings recorded. The sensor was left until the following day to stabilize and 3 measurements were collected every 5 min. The containers and sensors were covered with plastic bags to prevent evaporation.
10. Simultaneously to the data acquisition from the sensors, the water content of the soil was determined at each water content level by volumetric analysis. For this, a small cylinder of known volume (98 cm^3^) was removed and placed on pre-weighed and numbered trays. The trays were then placed in an oven at 105 °C and weighed daily until a constant weight was reached. At the end of the test, all the soil was returned to the original container (12 kg or 9 kg) where it was mixed outside the container by adding another quantity of water.

### 2.5. Statistical Analysis

Five statistical measures were computed to evaluate the accuracy and precision of the soil moisture sensors: the root mean square error (RMSE), the coefficient of determination (R^2^), the coefficient of variation (CV), the index of agreement (IA) and the mean bias error (MBE).

#### 2.5.1. The Root Mean Square Error (RMSE)

The RMSE is a frequently used measure of the difference between the value predicted by a model and the “real” value actually observed. The RMSE represents the sample standard deviation of the differences between predicted and observed values [31]. RMSE can be described by the following equation:

(7) $$\mathrm{RMSE}=\surd \frac{1}{N}\sum_{i=1}^{N}{\left(Mi-Pi\right)}^{2}$$

where N is the sample size, M is the measured (soil sample) value, and P is the predicted (sensor measurement) value. The units for the RMSE are volumetric water content (m^3^/m^3^).

#### 2.5.2. Coefficient of Determination (R²)

The R^2^ is used to evaluate how well a model explains or predicts future outcomes. The R^2^ can be defined as the percentage of the response variable variation that is explained by a model. Its value is given as a percentage (0–100%). A model fits the data well if the differences between predicted and observed values are small. The value of the R^2^ ranges between −1 and +1. The closer its value is to 1, the better the fit of the model to the variable we are trying to explain. Conversely, the closer to zero, the less well-fitted the model will be and, therefore, the less reliable it will be [30].

The coefficient of determination is usually denoted as R^2^, r^2^, or R-square, referring to the squared value of the correlation coefficient, r. The r is calculated through the following equation:

(8) $$R^{2}=\frac{\sum_{i=1}^{N}\left(P_{i}-\bar{P}\right)}{\sum_{i=1}^{N}{\left(M_{i}-\bar{M}\right)}^{2}}$$

where N is the sample size, M is the measured (soil sample) value, P is the predicted (sensor measurement) value, $\bar{M}$ is the average measured value and $\bar{P}$ is the average predicted value.

#### 2.5.3. The Index of Agreement (IA)

The IA reflects the degree to which observations are correctly estimated by the model. This IA was propused by Willmott, [32],as defined in Equation (9). The IA varies between 0 (complete mismatch between the value estimated by the model and the observed value) and 1 (a perfect fit).

(9) $$IA=1-\frac{\sum_{i=1}^{N}{\left(M_{i}-P_{i}\right)}^{2}}{\sum_{i=1}^{N}\left[{\left(P_{i}-\bar{M}\right)}^{2}+\left(M_{i}-\bar{M}\right)\right]}$$

where N is the sample size, M is the measured (soil sample) value, P is the predicted (sensor measurement) value, $\bar{M}$ is the average measured value.

#### 2.5.4. Mean Bias Error (MBE)

The MBE provides information on the nature of the error associated with the SM measured with the sensor with respect to the SM taken as the reference value. An MBE of zero implies that measured SM and reference SM are unbiased, while positive and negative values of MBE imply that measured SM is overestimating and underestimating reference SM, respectively [33]. The smaller the absolute value, the better the sensor performance.

(10) $$MBE=\frac{1}{N}\sum_{i=1}^{N}\left(Pi-Mi\right)$$

where N is the sample size, M is the measured (soil sample) value, and P is the predicted (sensor measurement) value.

#### 2.5.5. The Coefficient of Variation (CV)

The CV was determined to estimate the efficiency of the studied sensors. The CV was calculated through the following equation:

(11) $$\mathrm{CV}=(\frac{SD}{\bar{X}})*100$$

where $\bar{X}$ represents the mean of the sensor readings, and SD represents the deviations of the values from their arithmetic mean.

## 3. Results and Discussion

### 3.1. Evaluation of Factory Calibrated Moisture Sensors

The soil water content (θv) values measured by the sensors were compared with the results obtained by volumetric determination in the laboratory. Table 4 shows the performance evaluation of the different sensors calibrated with the manufacturer’s equation.

Accuracy is the ability of a sensor to estimate the true water content, while precision is an indication of the uniformity or repeatability of obtaining the same result [34]. Sensors need to have high accuracy and high precision.

For sensor accuracy, we used the RMSE (Equation (7)) and the R^2^ (Equation (8)) to check the quality of the fit.

Fares et al. [34] described the different categories for interpreting RMSE values: good (RMSE ≤ 0.01 m^3^/m^3^), fair (0.01 ≤ RMSE ≤ 0.05 m^3^/m^3^), poor (0.05 ≤ RMSE ≤ 0.10 m^3^/m^3^) and very poor (RMSE ≥ 0.10 m^3^/m^3^) Dong et al. [3] used the criterion of taking into account an R^2^ > 0.65 to assess sensor accuracy. The most accurate sensors were the HydraProbe and Teros 10, which had the highest R^2^ and lowest RMSE. The Teros 11, Drill & Drop and CS616 sensors showed a fair RMSE and the EnviroPro sensor a poor RMSE (RMSE = 0.07 m^3^/m^3^). Similar results were obtained by Dong et al. [3] when evaluating the performance of the CS616 and EC5 soil moisture sensors in laboratory tests and field. The performance of the factory calibrated CS616 and EC5 sensors did not meet the statistical criteria and the corrected equations obtained for both sensors improved the accuracy under field conditions. Lo et al. [35] analysed the inter-replicate variability of different soil sensors used for irrigation scheduling. Their results suggest that the Drill & Drop sensor has a higher inter-replicate variability than other sensors evaluated, such as the Watermark 200SS. This higher variability indicates that the values reported by different Drill & Drop units can differ significantly, compromising their reliability in accurately determining optimal irrigation timing. This behaviour could be attributed to the tendency of Drill & Drop to respond preferentially to wet areas within the soil profile, a phenomenon also observed in previous studies [36]. This indicates a need to improve the factory calibrations, especially for the EnviroProbe sensor. More accurate sensors provide more reliable data on soil water content, allowing irrigation to be adjusted in real time, helping farmers to determine when and how much to irrigate. This reduces the risk of under- or over-irrigation, improving both water use efficiency and crop health.

To evaluate the precision, the IA, MBE and CV were calculated to compare the results obtained from the sensor with the volumetric method. The range of IA is between 0 and 1, with a value of 0 indicating no agreement between measured and predicted values. A value of 1 indicates perfect agreement between observed and predicted values (Equation (9)) [32]. Varble and Chavez [37] set the criteria for MBE and RMSE to within ±0.02 m^3^m^−3^. Dong et al. [3] evaluated the various sensors studied according to the following criteria: RMSE < 0.035 m^3^/m^3^, IA > 0.8 and MBE ± 0.02 m^3^/m^3^. The MBE values for the Teros 11, HydraProbe and CS616 sensors underestimated the volumetric water content by an average of 0.0003 m^3^/m^3^, 0.0092 m^3^/m^3^ and 0.0072 m^3^/m^3^, respectively, with an IA > 0.94. In contrast, the Teros 10, EnviroPro and Drill & Drop sensors overestimated the volumetric water content by an average of 0.0101 m^3^/m^3^, 0.0574 m^3^/m^3^ and 0.0110 m^3^/m^3^, respectively. All probes have an MBE between ±0.02 m^3^/m^3^ and IA > 0.9, except the EnviroPro. The factory calibration of the EnviroPro was therefore not very accurate in the sandy soil (MBE = 0.0574 m^3^/m^3^ and IA = 0.786). The CV ranged from 2.129% for CS616 to 18.785% for Drill & Drop. Differences in CV values may be due to the specific electronics of each sensor, oscillation frequency, probe geometry, and their sensitivity to soil heterogeneity and air gaps [22].

**Figure 2:**
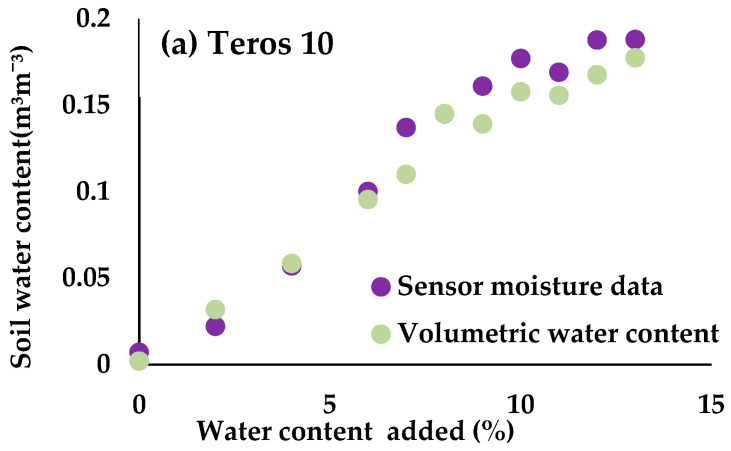

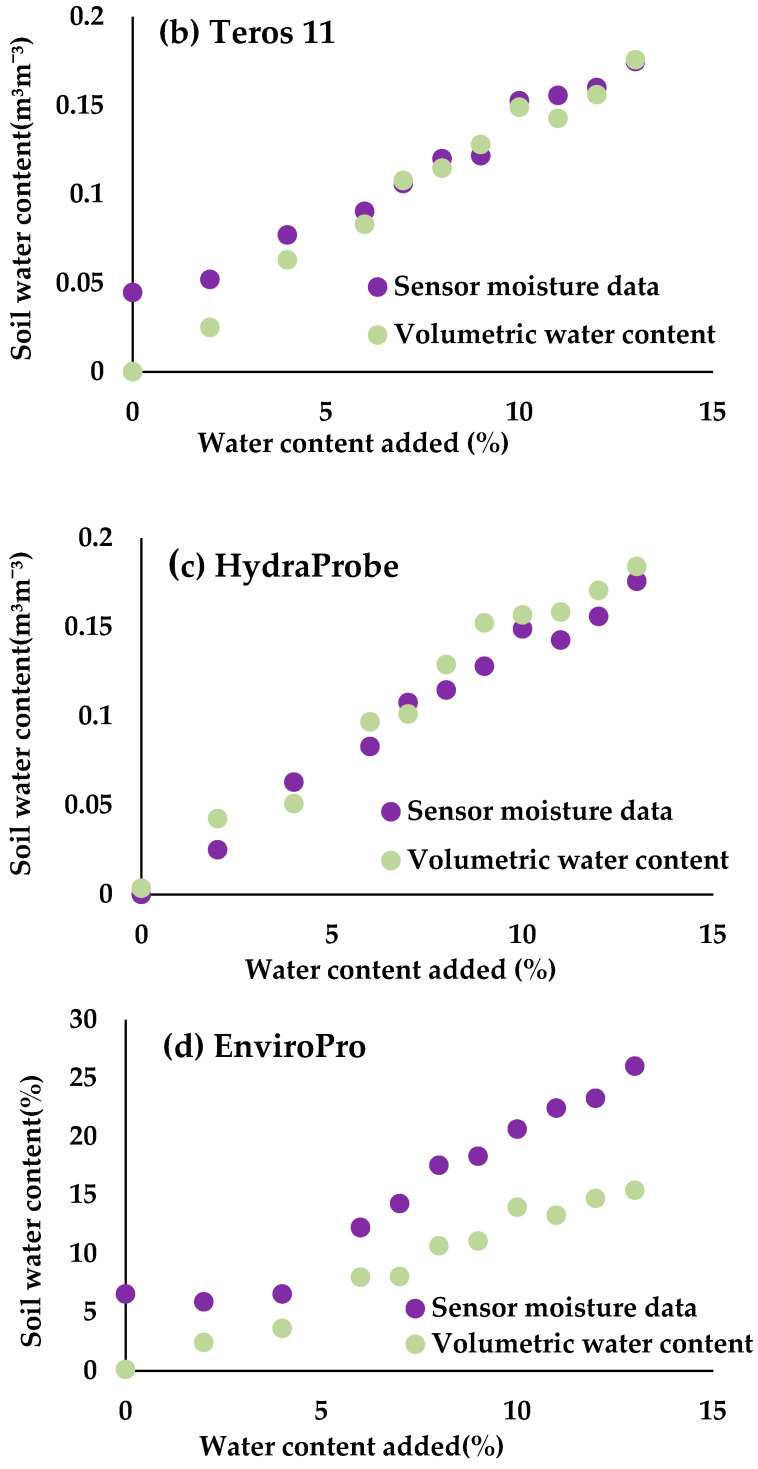

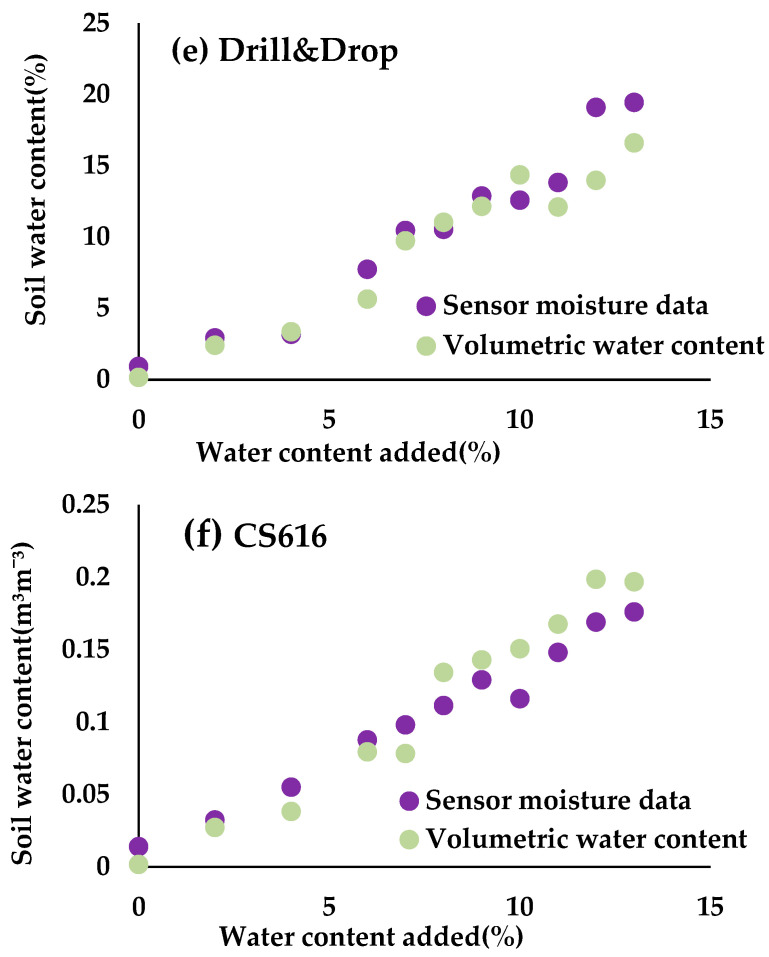
Distribution of volumetric moisture data and moisture sensor readings: (**a**) Teros 10, (**b**) Teros 11, (**c**) HydraProbe, (**d**) EnviroPro, (**e**) Drill & Drop and (**f**) CS616.

Figure 2 shows the soil moisture measured by the volumetric method and the moisture sensor readings. In Figure 2a, when the soil is completely dry, the moisture value obtained by the volumetric method is lower than the value recorded by the moisture sensor. As soon as a 2% volume of water is added to the total volume of the soil, the values are reversed and the values recorded by the moisture sensor are lower than those obtained by the volumetric method. As the moisture content of the soil increases (from 6% of the total soil water volume), the moisture values recorded by the sensors are higher than the moisture values obtained by the volumetric method. As in Figure 2b, the moisture values obtained with the sensor are higher than those obtained with the direct method throughout the calibration period, except when 9% volume of water is incorporated, where the values are reversed. When the soil is fully saturated, both methods give a very similar value for the soil water content. In Figure 2c, the moisture data recorded by the HydraProbe sensor underestimates the volumetric moisture content value. In the case of the EnviroPro sensor (Figure 2d), the values obtained by the sensor overestimate the values obtained by the volumetric method throughout the calibration period. In Figure 2e, when the soil is relatively dry, with small percentages of water volume in the incorporated soil, the water content values obtained by both methods are very similar. When adding 6% soil water volume, the Drill & Drop sensor readings overestimate the volumes obtained by the direct method, except for the time when 8% and 10% soil water is incorporated. For the CS616 sensor (Figure 2f), the values recorded by the sensor in the first phase of the calibration test overestimate the water content in the soil. After the incorporation of 8% water, the sensor starts to underestimate the soil water content compared to the direct method. This difference between the moisture sensor readings and those obtained by the volumetric method may be due to contact with the soil probe, high soil porosity, soils with water retention problems, etc. Walker et al. [38] carried out a calibration study of moisture sensors in loam soils. The results they obtained were a good approximation between the values obtained by the gravimetric method and the sensor readings, except under conditions of high porosity and high soil moisture content.

### 3.2. Evaluation of Moisture Sensors Calibrated for Sandy Soils

Some sensors require specific calibrations to better adapt to the soil conditions of the plot. If a sensor is calibrated for the local soil type, as in the case of sand in Doñana National Park, the readings will be more accurate, which in turn improves irrigation scheduling. A poorly calibrated sensor may underestimate or overestimate the moisture level, leading to incorrect irrigation decisions.

Correction equations for the sensors were developed (Table 5) based on the laboratory data. In terms of accuracy, the sensors had an R^2^ > 0.96 and the RMSE values ranged from 0.007 to 0.055 m^3^ m^−3^ (Table 5). The Teros 10, Teros 11, EnviroPro and Drill & Drop had a good RMSE [34]. The CS616 sensor also had an improved RMSE. However, the HydraProbe sensor had an increased RMSE in comparison to the factory equation.

In terms of precision, all sensors had increased IA and decreased MBE, except for the HydraProbe sensor which had a lower IA and a higher MBE. The CV was also lower for all sensors except the CS616 sensor. It can therefore be concluded that all sensors improved with the adjustments made to the factory calibration, except for the HydraProbe sensor which had a more accurate factory calibration for a sandy soil. Thus, the use of such sensors could facilitate their implementation in the field, reducing the time and costs associated with customised calibration.

### 3.3. Validation of Moisture Sensors with Sandy Soil Calibration

**Figure 3:**
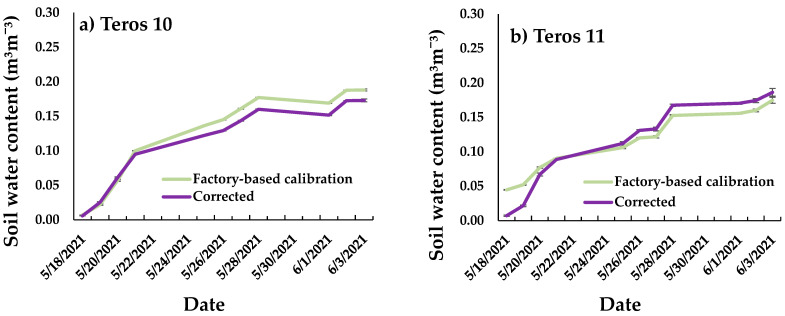

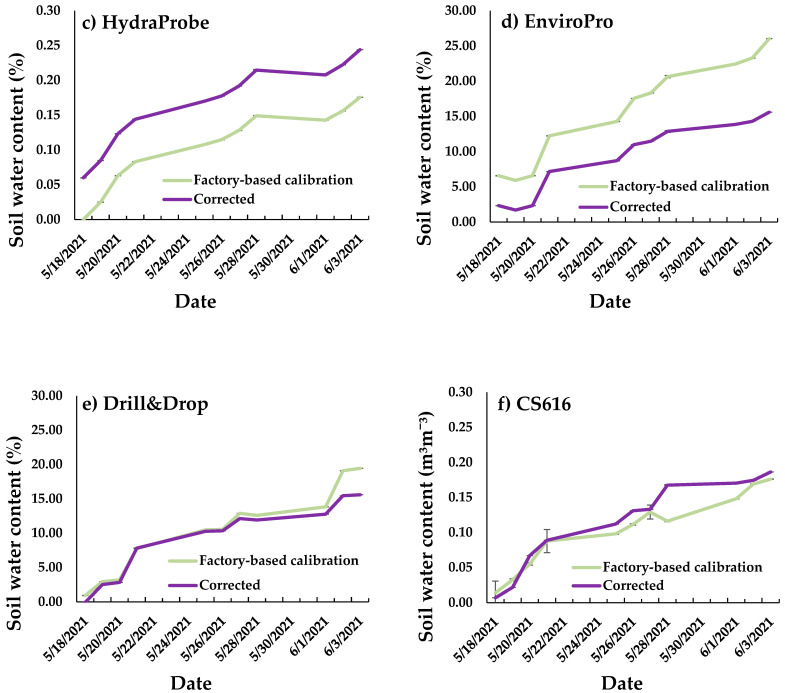
Comparison of factory calibration with corrected values for soil moisture sensors in sandy soils: (**a**) Teros 10; (**b**) Teros 11; (**c**) HydraProbe; (**d**) EnviroPro; (**e**) Drill & Drop; and (**f**) CS616. Error bars indicate the standard error.

Figure 3 shows the comparisons of the factory calibration of all the soil moisture sensors with the corrected values for sandy soil throughout the calibration cycle period from 18 May to 3 June of 2021. As can be seen in the different figures, moisture evolution is compared according to the values given by the factory calibration recommended by the manufacturer for this type of soil and the values corrected with the calibration obtained during the sensor calibration work.

The Teros 10 sensor (Figure 3a) shows similar behavior in the factory equation and the corrected equation under conditions of low soil moisture content. Above a soil moisture content of 0.09 m^3^/m^3^ there is an overestimation of the soil moisture values with the factory equation. This overestimation is similar between the values 0.10 m^3^/m^3^ and 0.19 m^3^/m^3^. Walker et al. [38] obtained similar results in a loam soil, where soil moisture sensor readings showed a good approximation to the values obtained by gravimetry except under conditions of high soil moisture content.

In the case of Teros 11 (Figure 3b), in contrast to Teros 10 there is an overestimation of the moisture values obtained with the factory equation at low moisture values between 0 m^3^/m^3^ and 0.04 m^3^/m^3^. Above these values, the two moisture curves are very similar, except for water contents close to saturation where the moisture values obtained with the factory equation underestimate the values obtained with the corrected equation.

From the factory equations of the Teros 10 and Teros 11 sensors (Figure 3a,b) it can be seen that there is a certain delay in the reading of the sensors when working with relatively low moisture content (almost dry soil). Initially, the Teros 10 sensor had a reading close to 0.01 m^3^/m^3^ with dry soil and the Teros 11 a reading of 0.045 m^3^/m^3^. When assessing the performance of the Teros family of sensors, it is important to note that the sensors have the same shape and operate at the same frequency. The main differences between the two sensors are that the Teros 11 sensor provides information on the temperature variable and the volume of measurement is larger (1010 cm^3^). These errors between readings obtained using the factory equation of one type of sensor and another may be due to the measurement volume [30].

For the HydraProbe sensors (Figure 3c), the soil moisture values obtained using the factory equation underestimated the moisture values obtained using the corrected equation. This discrepancy between one equation and the other is proportional for all moisture values. Better results in correlation indices were not always obtained when sensors were calibrated (Table 5), which could be due to possible errors in sample handling, limitations of instrument operation in low moisture conditions or even the presence of preferential pathways in the containers used.

The largest differences between the factory values and the corrected values are observed in the case of the EnviroPro probe (Figure 3d). In the case of the EnviroPro, in contrast to the HydraProbe, the factory values overestimate the actual soil moisture values obtained with the corrected equation. When the sandy soil is dry, the difference between the values obtained from the factory equation and the corrected equation is about 36%. When the sandy soil is wet, the difference between the values obtained using the two equations is much greater, around 60%. The installation technique of the soil moisture sensor plays a major role in sensor performance. Installation must be done with care, as the soil moisture sensor only measures a small volume of soil surrounding the sensor. It is essential that there is good contact between the sensor and the ground to avoid creating an air gap that can disturb the measurement [38,39,40]. Other authors have indicated that factors such as soil temperature and salinity can influence sensor readings [3] indicated that temperature fluctuations in soil are minimized as soil depth increases. In the case of the Drill & Drop sensor (Figure 3e), the sensor readings are similar to the values obtained with the corrected equation, except that there is an overestimation of the values obtained with the factory equation for soil moisture values close to saturation.

Finally, for the CS616 sensor (Figure 3f), when the soil is dry, the soil moisture values obtained from the factory equation and the corrected equation are similar. Around 25 May 2021, the soil moisture values obtained with the corrected equation overestimate the factory equation. This overestimation of the corrected equation values increases with increasing soil water content. Overall, the corrected equation for the CS616 sensor improved the accuracy of the sensor readings under high moisture conditions in a sandy soil field. Dong et al. [3] obtained similar results when evaluating the corrected equation for the CS616 sensor in a sandy soil field.

### 3.4. Tensiometers

**Figure 4:**
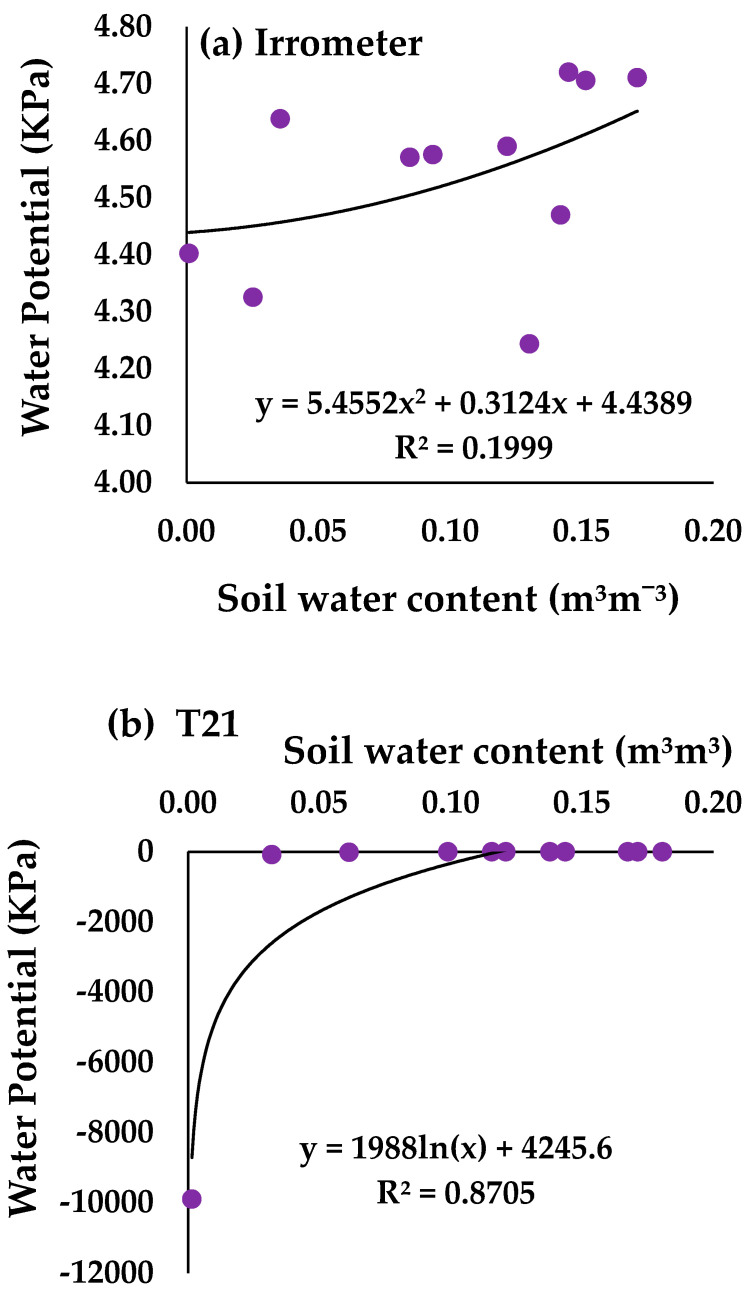

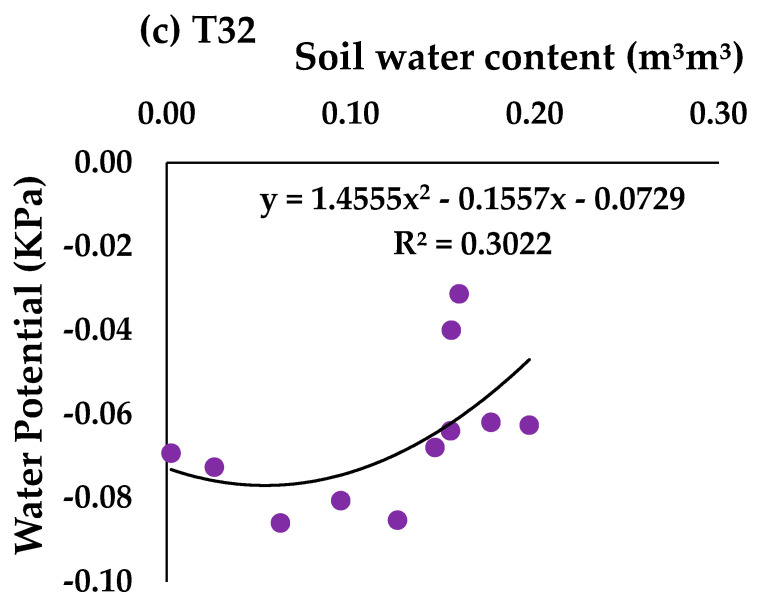
Relationship between soil water potential and volumetric water content in the soil: (**a**) Irrometer 10; (**b**) Teros 21; and (**c**) Teros 32.

Figure 4 shows the characteristic curve obtained with the tensiometer values and the volumetric water content in the soil. Dong et al. [3] indicated that a sensor was accurate when R^2^ > 0.65. The highest R^2^ was obtained with the Teros 21 sensor. In contrast, the Irrometer and Teros 32 tensiometers obtained very low R^2^ values. Tensiometers measure the soil water potential, which is a quantity directly related to the movement of water in the soil-plant-atmosphere system. These sensors have been used by many researchers to adjust irrigation scheduling [41,42]. However, tensiometers require constant maintenance to avoid or correct cavitation problems and poor contact between the porous capsule and the soil [9,43]. The Teros 21 sensor does not require maintenance, but both the Irrometer and the Teros 32 do. In addition, in the case of the Teros 32 sensor, the sensor must be maintained with deionized water. With regard to installation, the Teros 32 sensor must be installed at the correct angle, which is 45° according to the manufacturer’s specifications. In this work, it was observed that tensiometers require a longer stabilization time in the measurement than sensors that measure the water content in the soil. In this case, the tensiometer that took the longest time to stabilize was the Teros 32.

**Figure 5:**
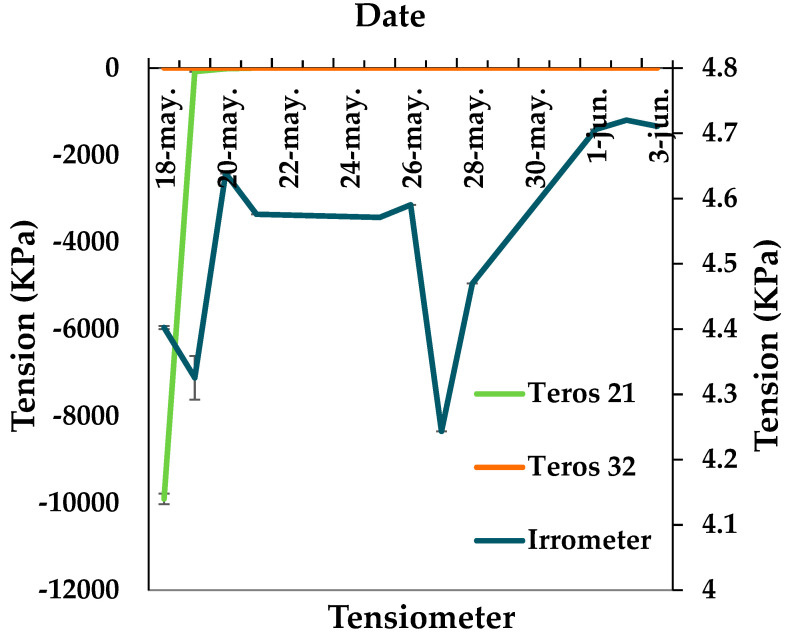
Evolution soil water potential in the soil: Irrometer 10, Teros 21 and Teros 32. Error bars indicate the standard error.

The soil water potential during the period from 18 May to 3 June can be seen in Figure 5. The values obtained with the Irrometer tensiometer range from 4.24 kPa to 4.37 kPa when the soil is dry to 4.71 KPa when the soil is fully saturated. Thus, there is minimal variation between the two values making it difficult to use this type of sensor for automatic irrigation scheduling. The same is true for the Teros 32 sensor, with the measuring range between −0.03 to −0.07 KPa. This range of values makes it very difficult to identify when the soil is dry or wet. With respect to the Teros 21 tensiometer, a change of water movement in the soil from −9990 KPa to −0.07 KPa is detected between 18 May 2021 and 19 May 2021, when the soil is practically dry. After 19 May 2021, the Teros 21 sensor detects no change in soil moisture. So, when the soil acquires a small percentage of moisture, these changes are not reflected in the sensor.

Table 6 provides a comparison of different commercial sensors based on the following variables: accuracy, experience of the consortium of companies participating in the LIFE4DOÑANA project with the sensor, expected robustness/warranty, ease of installation, price, measurement volume, measurement stabilization, and sensor-soil contact area. Each of the variables, depending on the sensor, was given a score from 0 to 100. The most accurate sensors were the Teros 10, as it had a higher R² and lower RMSE (Table 4). The consortium of companies had previously worked with the following sensors: Teros 10, Teros 11, HydraProbe, Drill & drop, CS616 and Irrometer. In contrast, they had no experience with the EnviroPro, Teros 21 and Teros 32 sensors. They wanted to reduce the number of sensors to be tested in this trial by using those that they had previously tested in other works, and which apparently worked well. The EnviroPro, Teros 21 and Teros 32 sensors were incorporated into this study because they wanted to expand the options of modular sensors and tensiometers. In terms of robustness, the sensors that appeared to be the most robust were the modular ones, as they were completely encapsulated by a PVC housing that protected them.

The CS616 sensor had two fairly long stainless steel rods. These sensors had the drawback that if the soil was too compact or if there was an obstacle such as a stone the rods were very prone to bending, hence providing an erroneous measurement value. In the case of the Irrometer and Teros 32 sensor, excessive care must be taken with the porous capsule as it can easily break. The sensors that presented the least difficulty in installation were the Teros 10, Teros 11, HydraProbe and Teros 21. Normally these types of sensors are installed with a barrier at the desired depth, avoiding the formation of preferential pathways in the soil and ensuring that the sensor-soil contact area is adequate. In modular sensors and the Irrometer sensor, it is important to place the sensor correctly in the container to obtain a correct reading, avoiding any movement in the containers as this can cause changes in the sensor-soil contact area, thereby affecting the recorded measurement. Any air pockets or excessive compaction around the sensor may influence the measurements taken and it is therefore important to avoid creating preferential water passage channels between the sensor and the soil volume in contact with it. The Teros 32 sensor requires more specialized labor for proper installation as, during the installation process, it is necessary to make sure that the angle formed with the soil is correct (45° as specified in the manual) and a certain level of maintenance is required (it must be filled with distilled water). Sensors such as the Teros 10, Teros 11, HydraProbe and Teros 21, which are easy to install and require little maintenance, can influence irrigation scheduling by making monitoring more efficient. In contrast, sensors that require constant maintenance, such as some tensiometers, can cause delays in irrigation scheduling due to the time required for maintenance, making them difficult to integrate into automated irrigation systems.

In terms of price, the cheapest sensors were the Teros 10 and the CS616. Typically, the use of more durable and accurate components contributes to a higher sensor acquisition cost [44]. Compared to other sensors, the Teros 10 sensor provides us with fairly accurate information at a relatively low acquisition cost. The sensor with the largest measurement volume was the Teros 11, which measures at a single depth. However, if using modular sensors that measure the water content in the soil at different depths, the EnviroPro has the largest measurement volume. These modular sensors are particularly useful for crops that require precise monitoring in multiple soil layers. This allows for more detailed irrigation scheduling, as irrigation can be based on the moisture requirements of different areas of the soil profile. The time it took for the sensor to take reliable measurements from the time it was installed (stabilization of the measurement) was very fast for sensors that measure soil water content. However, the tensiometers needed some time to stabilize the measurement from the moment they were installed. The Teros 32 sensor needed about 3.5 h to take reliable measurements from installation, the Teros 21 about 3 h and the Irrometer about 2 h. So that, sensors with rapid stabilisation of readings could allow more immediate adjustment of irrigation in response to soil conditions, optimising water use.

With regard to the sensor-soil contact area, proper placement and stabilization within the container are crucial for the modular sensors to obtain accurate readings. Movement or displacement of the sensor can lead to variations in the sensor-soil contact area, which in turn affects the recorded measurements. Air pockets can disrupt the uniformity of soil contact, leading to inaccurate measurements. The soil must be carefully compacted around the sensor to ensure good contact without being overly compressed, which could alter its natural properties. Excessive compaction carried out around the sensor can affect the soil’s physical properties and thus the sensor readings. The aim should be for a natural compaction level similar to that of the surrounding soil environment.

## 4. Conclusions

This study highlights the importance of selecting sensors based on specific needs such as accuracy, cost, robustness, and ease of installation. Proper calibration and maintenance practices are essential to ensure reliable soil moisture readings that influence farmers’ ability to optimise water use and improve irrigation efficiency, contributing to more sustainable and productive crop management.

1. Accuracy and cost: The Teros 10 and CS616 sensors were the most cost-effective options while still providing reasonably accurate measurements. The Teros 10 sensor offered high accuracy at a relatively low acquisition cost.
2. Measurement volume: the Teros 11 had the largest measurement volume for a single depth sensor. Among the modular sensors, the EnviroPro measured the largest volume of soil water content at various depths.
3. Stabilization time: Sensors measuring soil water content stabilized quickly after installation. In contrast, tensiometers required a longer stabilization period.
4. Sensor-soil contact: Proper placement and stabilization of sensors are crucial for accurate readings. Movement or displacement can cause variations in the sensor-soil contact area, leading to inaccurate measurements. Careful compaction around the sensor is necessary to avoid air pockets and ensure good contact without altering the soil’s natural properties.
5. Ease of installation and robustness: The Teros 10, Teros 11, and HydraProbe sensors were the easiest to install. Modular sensors were deemed the most robust due to their encapsulated design, while the CS616 sensor’s stainless steel rods posed challenges in compact or obstacle-laden soils.
6. Sensor maintenance: Tensiometers, such as the Teros 21, require minimal maintenance compared to others like the Irrometer and Teros 32, which need constant attention to avoid cavitation and ensure proper contact between the porous capsule and soil.
7. Future Directions: It would be valuable to investigate the durability and consistency of sensors under varying environmental conditions, such as changes in temperature, salinity and soil compaction, which could affect measurements over time. Such studies could validate the performance of the sensors in long-term use scenarios and under different field conditions. Moreover, the automation of irrigation is becoming more and more important in agriculture, moisture sensors could be integrated with smart irrigation systems to optimise water use in real time. Exploring this integration would allow the development of irrigation algorithms that automatically adjust irrigation based on real-time sensor data, promoting more sustainable and efficient agriculture.
8. Given that some sensors, such as the Teros 32 and Irrometer tensiometers, require specialised manpower for correct installation and maintenance in the field, it would be desirable for the company supplying the sensors to provide basic sensor training for farmers and agricultural technicians.

## Figures and Tables

**Table 1:** Features of the soil moisture sensors.

Photo	Manufacturer	Measuring Technique	Sensor Model	Measures	Sensor Outputs	Sensing Volume	Soil Moisture Range
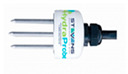	Stevens Water, Portland, OR, USA	Impedance (I)	HydraProbe II	Soil moisture (m^3^/m^3^) Soil salinity (S/m) Soil temperature (°C)	Ꜫ, Ꜫ’, EC, T	V = 40 cm^3^	0.00–1
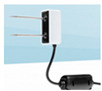	Decagon, Munich, Germany	FDR	Teros 10	Soil moisture (m^3^/m^3^)	Voltage	V = 430 cm^3^	0.00–0.64
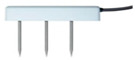	Decagon, Munich, Germany	FDR	Teros 11	Soil moisture (m^3^/m^3^) Soil temperature (°C)	Voltage	V = 1010 cm^3^	0.00–0.70
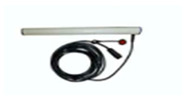	Sentek, Stepney, Australia	FDR	Drill & Drop	Volumetric soil moisture content (%), Soil salinity(dS/m), Soil temperature (°C)	Voltage	V = 102.4 cm^3^	0–100
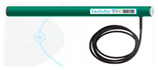	Entelechy realisin potential, Golden Glove, Australia	FDR	EnviroPro	Volumetric soil moisture content (%), Soil salinity (dS/m) Soil temperature(°C)	Voltage	V = 353.69 cm^3^	0–50
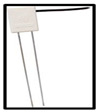	Campbell Scientific, Logan, UT, USA	TDR	CS616	Volumetric soil moisture content (%)	Period	V = 241 cm^3^	0–50

Where measures real (Ꜫ) and imaginary (Ꜫ’) dielectric constant; EC is electrical conductivity; T is the temperature. Period is in microseconds.

**Table 2:** Features of the tensiometers.

Photo	Manufacturer	Sensor Model	Measures	Range	Resolution	Accuracy
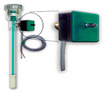	IRROMETER (Riverside, CA, USA)	RSU-C-34	4–20 mA loop current	0 to 34 kPa (0–34 cb)		±0.5%
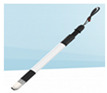	METER (UMS) (Hammond, LA, USA)	Teros 32	Soil water potential (kPa) Temperature (°C)	−85 to +50 kPa	0.0012 kPa	±15 kPa
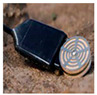	METER (UMS)	Teros 21	Soil water potential (kPa) Temperature (°C)	−9 to −100,000 kPa	0.1 kPa	±(10% of the reading +2 kPa) from −9 to −100 kPa

**Table 3:** Parameters obtained with HYPROP + WP4C.

Parameter	Soil Water Content
Field capacity	12.40 (Vol.%)
Permanent wilting point	4.70 (Vol.%)
Water available to the plant	7.70 (Vol.%)

**Table 4:** Statistical analysis to compare measured values to sensor values.

Measuring Technique	Sensor Model	R^2^	RMSE	IA	MBE	CV (%)
FDR	Teros 10	0.980	0.015	0.970	0.0101	9.714
FDR	Teros 11	0.970	0.019	0.941	−0.0003	17.626
Impedance (I)	HydraProbe	0.969	0.014	0.989	−0.0092	9.879
FDR	EnviroPro	0.954	0.070	0.786	0.0574	16.422
FDR	Drill & Drop	0.918	0.021	0.933	0.0110	18.785
TDR	CS616	0.963	0.020	0.941	−0.0072	2.129

R^2^ is the coefficient of determination; RMSE is the root mean square error; IA is the index of agreement; MBE is the mean bias error; and CV is the coefficient of variation.

**Table 5:** Correction equations for soil moisture sensors.

Sensor Model	Corrected Equation	R^2^	RMSE	IA	MBE	CV (%)
Teros 10	θv = 23.6 × (θvi)^3^ − 7.3701 × (θvi)^2^ + 1.4928 × (θvi) − 0.0043	0.997	0.007	0.993	0.0000	6.677
Teros 11	θv = −4.8542 × (θvi)^2^ + 2.4433 × (θvi) − 0.0924	0.984	0.007	0.993	0.0000	6.497
HydraProbe	θv = 0.3946 × (θvi)^2^ + 0.9786 × (θvi) + 0.006	1.000	0.055	0.675	0.0540	7.373
EnviroPro	θv = −0.0125 × (θvi)^2^ + 1.0896 × (θvi) − 4.2973	0.991	0.010	0.981	−0.0110	10.974
Drill & Drop	θv = −0.0265 × (θvi)^2^ + 1.3843 × (θvi) − 1.3192	0.968	0.012	0.990	−0.0047	13.480
CS616	θ = 0.5798 × (θvi)^2^ + 1.1673 × (θvi) − 0.0178	1.000	0.019	0.957	0.0000	12.493

R^2^ is the coefficient of determination; RMSE is the root means square error; IA is the index of agreement; MBE is the mean bias error; CV is the coefficient of variation; θvi is the value from the factory-based calibrated sensor; and θv is the corrected value.

**Table 6:** Comparison of different sensors.

Sensor	Teros 10	Teros 11	HydraProbe	EnviroPro	Drill & Drop	CS616	Teros 21	Teros 32	Irrometer
Precision	90	90	70	90	90	80	90	30	20
Consortium experience with the probe	90	90	90	0	80	90	80	0	90
Expected robustness/guarantee	80	80	80	90	80	50	80	30	30
Ease of installation	80	80	80	80	80	70	80	40	50
Price (Euro)	100	90	50	80	90	100	90	60	80
Measuring volume (cm^3^)	80	90	40	70	50	60	50	10	10
Stabilization of the measure	100	100	100	100	100	100	100	20	30
Sensor-soil contact area	100	100	100	80	80	70	80	80	80
**Valuation**	**90**	**90**	**76.25**	**73.75**	**81.25**	**77.5**	**81.25**	**33.75**	**48.75**

## Data Availability

Data are contained within the article.
